# Ventilation Assessment by Carbon Dioxide Levels in Dental Treatment Rooms

**DOI:** 10.1177/00220345211014441

**Published:** 2021-05-11

**Authors:** Q. Huang, T. Marzouk, R. Cirligeanu, H. Malmstrom, E. Eliav, Y.-F. Ren

**Affiliations:** 1 Eastman Institute for Oral Health, University of Rochester Medical Center, Rochester, NY, USA

**Keywords:** indoor air quality, pathogen transmission, air filter, baking soda, dentistry, COVID-19

## Abstract

It is important for dental care professionals to reliably assess carbon dioxide (CO_2_) levels and ventilation rates in their offices in the era of frequent infectious disease pandemics. This study was to evaluate CO_2_ levels in dental operatories and determine the accuracy of using CO_2_ levels to assess ventilation rate in dental clinics. Mechanical ventilation rate in air change per hour (ACH_VENT_) was measured with an air velocity sensor and airflow balancing hood. CO_2_ levels were measured in these rooms to analyze factors that contributed to CO_2_ accumulation. Ventilation rates were estimated using natural steady-state CO_2_ levels during dental treatments and experimental CO_2_ concentration decays by dry ice or mixing baking soda and vinegar. We compared the differences and assessed the correlations between ACH_VENT_ and ventilation rates estimated by the steady-state CO_2_ model with low (0.3 L/min, ACH_SS30_) or high (0.46 L/min, ACH_SS46_) CO_2_ generation rates, by CO_2_ decay constants using dry ice (ACH_DI_) or baking soda (ACH_BV_), and by time needed to remove 63% of excess CO_2_ generated by dry ice (ACH_DI63%_) or baking soda (ACH_BV63%_). We found that ACH_VENT_ varied from 3.9 to 35.0 in dental operatories. CO_2_ accumulation occurred in rooms with low ventilation (ACH_VENT_ ≤6) and overcrowding but not in those with higher ventilation. ACH_SS30_ and ACH_SS46_ correlated well with ACH_VENT_ (*r* = 0.83, *P* = 0.003), but ACH_SS30_ was more accurate for rooms with low ACH_VENT_. Ventilation rates could be reliably estimated using CO_2_ released from dry ice or baking soda. ACH_VENT_ was highly correlated with ACH_DI_ (*r* = 0.99), ACH_BV_ (*r* = 0.98), ACH_DI63%_ (*r* = 0.98), and ACH_BV63%_ (*r* = 0.98). There were no statistically significant differences between ACH_VENT_ and ACH_DI63%_ or ACH_BV63%_. We conclude that ventilation rates could be conveniently and accurately assessed by observing the changes in CO_2_ levels after a simple mixing of household baking soda and vinegar in dental settings.

Carbon dioxide (CO_2_) level is an important indicator of ventilation in occupied indoor environments. CO_2_ is a by-product of human metabolism and exists in high levels in exhaled air. Atmospheric CO_2_ level is approximately 400 parts per million (ppm) in outdoor environments, but CO_2_ in human exhaled air reaches on average 40,000 ppm in concentration (Issarow et al. 2015). CO_2_ level is therefore often used as a proxy for indoor air quality as well as a risk marker for transmission of airborne diseases since it is inert, its indoor emission source (human) is known, and its measurement is inexpensive and accurate (Batterman 2017). Increased CO_2_ level is often associated with poor ventilation and overcrowding. Accumulation of CO_2_ occurs concurrently with accumulation of respiratory pathogens in a room where an infected person is present but not wearing a mask, which may increase the risk of disease transmission. High levels of CO_2_ have long been associated with the transmission of infectious respiratory diseases such as tuberculosis, influenza, and rhinovirus infections (Myatt et al. 2004; Richardson et al. 2014; Wood et al. 2014). Indoor CO_2_ levels have therefore been widely used to model the risks of airborne infectious disease transmission (Rudnick and Milton 2003; Issarow et al. 2015; Harrichandra et al. 2020), including that of coronavirus infection transmission in dental offices (Zemouri et al. 2020).

Clinical spaces with good ventilation should have CO_2_ levels close to that of outside air at approximately 400 ppm (ASHRAE 2007; Issarow et al. 2015). Higher CO_2_ levels indicate poor ventilation, accumulation of exhaled air, and increase in the fraction of “rebreathed air” in the indoor environment, which is proven to be a risk factor for infectious disease transmissions (Rudnick and Milton 2003; Richardson et al. 2014; Wood et al. 2014; Issarow et al. 2015). CO_2_ levels have been used to estimate ventilation rates in dental offices (Godwin et al. 2003; Helmis et al. 2007; Helmis et al. 2008). Ventilation rate was found to be 1.12 air change per hour (ACH) in a typical dental clinic in the United States (Godwin et al. 2003) and was on average 5 ACH in a dental school clinic in Greece where the doors and windows were opened for cross-ventilation (Helmis et al. 2007). Both studies used natural buildup of CO_2_ levels in dental clinics to estimate the ventilation rate through mathematic models, but none actually verified the ventilation estimates using standard methodologies such as high-precision airflow sensors (ASHRAE 2017). It is not known if these estimates were accurate as both methods require mathematical modeling based on several assumptions related to CO_2_ generation, buildup, and dispersion over a relatively lengthy period of time, which may result in erroneous estimates if any of the assumed conditions are not met (Batterman 2017). A simpler and more reliable method is needed if CO_2_ level is to be used by dental care professionals to assess the ventilation rate of their treatment rooms.

The purpose of the present study was 2-fold: 1) to evaluate CO_2_ level and its associated factors in dental operatories and 2) to determine the accuracy of various methods using CO_2_ levels to assess ventilation rate in dental clinics. Our aim was to find a practical tool that will enable dental care professionals to conveniently and accurately monitor CO_2_ levels and assess the ventilation rates in order to devise a pragmatic and effective strategy for ventilation improvement in their work environment.

## Methods

### Study Settings

We conducted the CO_2_ concentration and ventilation rate assessments in 10 closed treatment rooms ranging from 667 to 1,221 cubic feet (ft^3^) in size in a multifloor building in an academic dental institute. Mechanical ventilation of the rooms was provided by 3 air handlers that drew 60% outside air to the ventilation system.

### Determining Room Airflow and Mechanical Ventilation Rates

Mechanical ventilation in air change per hour (ACH_VENT_) was measured with an air velocity sensor integrated in an airflow balancing hood (ADM-850L Airdata Multimeter with CFM-850L FlowHood; Shortridge Instruments) as described elsewhere (Ren et al. 2021).

### Assessing CO_2_ Levels during Dental Treatment Procedures

We measured CO_2_ levels in 2 dental treatment rooms when dental procedures were performed. The 2 rooms represented 2 extremes in ventilation rates, with one at 3.9 air change per hour and the other at 35. The number of persons in the rooms was recorded in real time when a person was entering and leaving the room. CO_2_ levels were measured at a 1-min interval using a consumer-grade CO_2_ sensor (Aranet4, range 0–9,999 ppm, accuracy ±50 ppm; SAF Tehnika).

### 24-h Continuous Monitoring CO_2_ in Dental Treatment Rooms

To further explore the dynamics of CO_2_ levels in dental treatment rooms throughout the day and assess accuracies of the steady-state models of CO_2_ for ventilation assessments, we continuously measured the CO_2_ levels in 10 dental treatment rooms for 24 h and recorded the procedures performed and number of persons in the room.

### Assessing Ventilation Rate by Natural CO_2_ Level Modeling in Dental Treatment Rooms

We used the steady-state model described by Batterman (2017) to calculate the air change rate of treatment rooms and compared the outcomes with that of measured mechanical ventilation.

The steady-state air change rate (ACH_SS_) is calculated as follows (Batterman 2017):

(1) $$ACH_{SS}=\;6\;\times \;10^{4}n\;Gp\;/\;\left[V\left(C_{SS}-C_{R}\right)\right],$$

where *n* = number of persons in the room, G_P_ = average CO_2_ generation rate, V = volume of the room in cubic meters (m^3^), C_SS_ = steady-state indoor CO_2_ level in ppm, and C_R_ = CO_2_ level in outdoor air in ppm.

The CO_2_ generation rate G_P_ is affected by many factors and may vary by human activity, physical size, sex, and race (Qi et al. 2014; Persily and de Jonge 2017). G_P_ of 0.46 L/min or 0.30 L/min was used in previous studies to represent CO_2_ generation (Godwin et al. 2003; Batterman 2017). As CO_2_ generation rates and activity levels by dental care providers and their patients are unknown and may not be constant, we used both values to calculate the air change rate ACH_SS_ and assess the correlations between ACH_SS_ and ACH_VENT_ at 2 G_P_ levels.

### Assessing Ventilation Rates by CO_2_ Decays Using Dry Ice

Ventilation rates by CO_2_ clearance using dry ice (ACH_DI_) were determined as described by Batterman (2017) using CO_2_ concentration decays:

(2) $$ACH_{DI}=\;1/\Delta t\;ln\left[\left(C_{1}-\;C_{R}\right)/\left(C_{0}-\;C_{R}\right)\right],$$

where Δt = period between measurements, C_0_ and C_1_ = CO_2_ levels measured at the beginning and the end of the decay period (ppm), and C_R_ = CO_2_ level in outdoor air (ppm).

### Assessing Ventilation Rates by CO_2_ Decays Using Baking Soda

Considering that dental care professionals in private practices may not have ready access to dry ice, we developed a method to rapidly generate CO_2_ in dental offices using baking soda (Arm & Hammer Pure; Church & Dwight) and vinegar (Heinz all-natural distilled white vinegar) (see Appendix for detailed protocol). Mixing baking soda (NaHCO_3_) with vinegar containing 5% acetic acid (CH_3_COOH) will generate CO_2_ as follows:

(3) $$NaHCO_{3}+CH_{3}COOH\to CH_{3}COONa+H_{2}O+CO_{2}.$$

Ventilation rates by CO_2_ clearance using baking soda and vinegar (ACHBV) could then be calculated using Equation 2 as above.

### Estimating Ventilation Rate by Time to 63% Removal of Excess CO_2_

Based on a commonly used formula for rate of purging airborne contaminants, 1 complete air change will replace 63% of airborne contaminants in the room with outdoor air (Nardell et al. 1991; Fernstrom and Goldblatt 2013; Jimenez 2020). Ventilation rate can therefore be simply calculated using the time needed to reach a 63% reduction of excess CO_2_ from its peak level:

(4) $$ACH_{T63\%}=\;60/\left(t_{2}-t_{1}\right),\;with\;t_{1}=\;0,$$

where t_1_ = initial time point with indoor CO_2_ at peak level, and t_2_ = time point (min) when excess CO_2_ is reduced by 63%. Indoor CO_2_ at peak level (C_S_) is the sum of outdoor CO_2_ (C_R_) and excess CO_2_ (C_E_) generated by dry ice or baking soda. As CO_2_ measurement starts at peak level, t_1_ is therefore always 0. Time needed to remove 63% C_E_, or t_2_, is the time point when indoor CO_2_ level is at C_63%E_ = C_S_ – 63% C_E_, where C_E_ = C_S_ – C_R_.

### Statistical Analysis

We performed multiple regression analysis using CO_2_ levels as the dependent variable and number of persons in the room, ventilation rate, room size, and outdoor CO_2_ level as independent variables. We analyzed the dynamics of CO_2_ levels during dental treatment procedures using descriptive analysis and compared the steady-state CO_2_ levels between rooms with poor and good ventilation using *t* tests. Mechanical ventilation rate was compared with air change rates calculated with different methods based on CO_2_ levels to assess the correlation (Pearson’s *r*) and differences (paired *t* test) between the 2 methods of ventilation assessments.

## Results

### Mechanical Ventilation Rate of the Dental Treatment Rooms

The volumetric sizes, airflow rates, and ventilation rates of the rooms are presented in Table 1. The rooms are on average 882 ft^3^ in volume (range 667–1,221 ft^3^). Air change rate by ventilation varied from 3.9 to 35.0 with a mean of 13.2 ± 10.6 per hour.

**Table 1.** Volumetric Sizes and Mechanical Ventilation Rates of Dental Treatment Rooms.

Room No.	Volume, ft^3^	SAF, ft^3^/min	EAF, ft^3^/min	ACH_S_	ACH_E_	ACH_VENT_	Floor
002	815	82	27	6.0	2.0	6.0	0
003	787	69	27	5.3	2.1	5.3	0
008	1,221	149	103	7.3	5.1	7.3	2
012	1,015	152	64	9.0	3.8	9.0	2
019	686	59	400	5.2	35.0	35.0	1
021	861	75	51	5.3	3.6	5.3	1
022	833	55	46	3.9	3.3	3.9	1
031	962	337	210	21.0	13.1	21.0	2
032	667	289	220	26.0	19.8	26.0	2
033	970	211	220	13.1	13.6	13.6	2
Mean	882	148	137	10.2	10.1	13.2	N/A
SD	166	101	122	7.6	10.6	10.6	N/A

ACH_E_, air change per hour based on exhaust airflow rate; ACH_S_, air change per hour based on supply airflow rate; ACH_VENT_, air change per hour based on mechanical ventilation; EAF, exhaust airflow rate in cubic feet per minute; N/A, not applicable; SAF, supply airflow rate in cubic feet per minute.

### CO_2_ Levels during Dental Treatment Procedures

**Figure 1.**
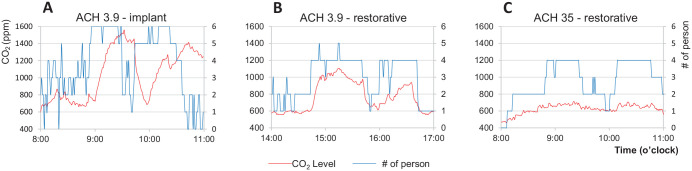
Carbon dioxide (CO_2_) levels during dental treatment procedures in operatories with low and high mechanical ventilation. (**A**) Significant CO_2_ accumulation occurred in a room with low ventilation (air change per hour [ACH] = 3.9) and multiple persons in the room during clinical teaching activities for dental implant surgery. CO_2_ level is associated with ventilation rate in rooms with the same number of persons. Peak CO_2_ level reached 1,100 ppm in the room with 3.9 ACH (**B**) but stayed under 700 ppm in the rooms with 35 ACH (**C**).

As shown in Figure 1, CO_2_ levels were significantly higher in the room with low ventilation (less than 4 air change per hour) and reached nearly 1,600 ppm when 6 persons were in the room. The increased number of persons was related to teaching activities involving dental implant surgery where additional graduate students were allowed to observe the procedures. Comparing the 2 rooms with the same number of persons for the same restorative procedures, CO_2_ levels reached 1,100 ppm at the peak in the room with 3.9 air change per hour but stayed below 700 ppm in the room with 35 air change per hour (*P* < 0.0001). CO_2_ accumulation appeared to be associated with crowding and low ventilation rate.

### Continuous CO_2_ Monitoring in Dental Treatment Rooms

**Figure 2.**
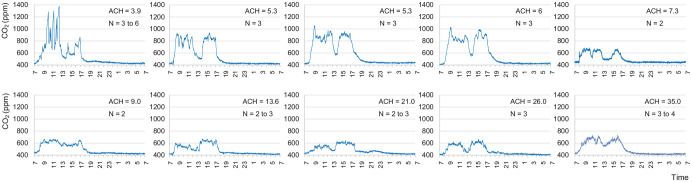
The 24-h continuous measurements of carbon dioxide (CO_2_) levels in 10 dental treatment rooms with various ventilation rates. CO_2_ accumulation occurred in rooms with lower ventilation rates (air change per hour [ACH] ≤6). CO_2_ levels stayed under 800 ppm in rooms with a higher ventilation rate and lower number of persons. CO_2_ level in nonworking hours is close to that of outdoors at 400 ppm in all rooms.

We continuously monitored the CO_2_ levels for 24 h in the 10 treatment rooms. The dental procedures included exams, hygiene, extractions, restoratives, endodontics, dental implant surgery, and periodontal surgery. Number of persons in the rooms varied from 2 to 6, with more people in the room during dental implant surgeries. The CO_2_ levels in early morning (5:00–7:00 a.m.) were at a level of 421 ± 10 ppm, similar to outdoor levels (413 ± 15 ppm) (Fig. 2). The steady-state CO_2_ level (C_SS_) during dental procedures, which is the mean concentration of CO_2_ at the plateau level when the number of persons in the room stays unchanged for at least 5 min, ranged from 543 ppm to 1,374 ppm (786 ± 207 ppm) (Appendix Table 1). Multiple regression analysis showed that CO_2_ levels were significantly correlated to the number of persons in the room (β = 90.2, *P* = 0.006), ventilation rate (β = 11.0, *P* = 0.001), and volumetric size of the room (β = −0.50, *P* = 0.049) but not to outdoor CO_2_ levels (β = 4.15, *P* = 0.160).

As shown in Figure 2, CO_2_ levels in rooms with more than 6 air change per hour rarely reached 800 ppm. In rooms with less than 6 air change per hour, however, the CO_2_ levels were consistently greater than 800 ppm and approached 1,400 ppm in a room with 3.9 air change per hour when number of persons in the room increased.

### Ventilation Rates by Steady-State CO_2_ Level Modeling

Ventilation rates with CO_2_ generation at 0.30 L/min (ACH_SS30_) and 0.46 L/min (ACH_SS46_) were calculated using equation (1) for 2 dental procedures in each room (Appendix Table 1). Both ACH_SS30_ and ACH_SS46_ were similarly correlated with ACH_VENT_ (*r* = 0.83, *P* = 0.003). ACH_SS30_ approximated closely to mechanical ventilation rates in rooms with less than 6 air change per hour (mean difference = −0.6, paired *t* = −1.24, *P* = 0.304) but significantly underestimated those in rooms with greater than 6 air change per hour (mean difference = −8.7, paired *t* = −2.59, *P* = 0.049). The opposite is true for ACH_SS46_; it significantly overestimated the ventilation rates in rooms with 6 air change per hour or less (mean difference = 3.7, paired *t* = 6.78, *P* = 0.007) but approximated closer to those in rooms with greater than 6 air change per hour (mean difference = −3.5, paired*t* = −1.14, *P* = 0.307) (Table 2).

**Table 2.** Comparisons between Mechanical Ventilation Rates and Ventilation Rates Estimated from Natural CO_2_ Levels and CO_2_ Released by Dry Ice or Baking Soda.

Room No.	ACH_VENT_	ACH_SS30_	ACH_SS46_	ACH_DI_	ACH_BV_	ACH_DI63_	ACH_BV63_
002	6.0	5.4	8.2	5.6	6.1	5.6	6.1
003	5.3	6.1	9.5	4.8	4.7	4.9	4.9
008	7.3	4.9	7.5	5.2	6.9	6.7	6.8
012	9.0	6.1	9.3	9.2	9.2	9.2	9.4
019	35.0	11.0	16.8	28.5	27.0	30.8	27.3
021	5.3	5.9	9.0	4.4	4.8	4.4	5.3
022	3.9	5.6	8.6	3.6	4.8	4.1	4.6
031	21.0	11.2	17.2	17.6	16.3	17.2	16.7
031	26.0	16.3	24.9	19.0	22.5	17.3	22.2
033	13.6	10.2	15.5	14.3	15.8	14.3	16.2
Mean	13.2	12.6	8.3	11.2	11.8	11.5	11.9
SD	10.6	5.7	3.7	8.4	8.1	8.2	8.1

ACH_BV_, ventilation rate estimate by carbon dioxide (CO_2_) decay constants using baking soda and vinegar; ACH_BV63_, ventilation rate estimate by time needed to remove 63% excess CO_2_ by baking soda and vinegar; ACH_DI_, ventilation rate estimate by CO_2_ decay constants using dry ice; ACH_DI63_, ventilation rate estimate by time needed to remove 63% excess CO_2_ by dry ice; ACH_SS30_, ventilation estimate by steady-state CO_2_ level with CO_2_ generation at 0.3 L/min per person; ACH_SS46_, ventilation estimate by steady-state CO_2_ level with CO_2_ generation at 0.46 L/min per person; ACH_VENT_, mechanical ventilation rate.

As ventilation rate is likely below 6 air change per hour in private dental practices in small freestanding buildings in the United States (Godwin et al. 2003), CO_2_ generation rate (G_P_) of 0.30 L/min is more appropriate for ventilation estimates using equation (1). We list the corresponding CO_2_ levels and ventilation rates in Appendix Table 2. Dental care professionals may use the CO_2_ levels measured in their treatment rooms to roughly estimate the ventilation rate using this table.

### Ventilation Rates by CO_2_ Decays Using Dry Ice or Baking Soda

**Figure 3.**
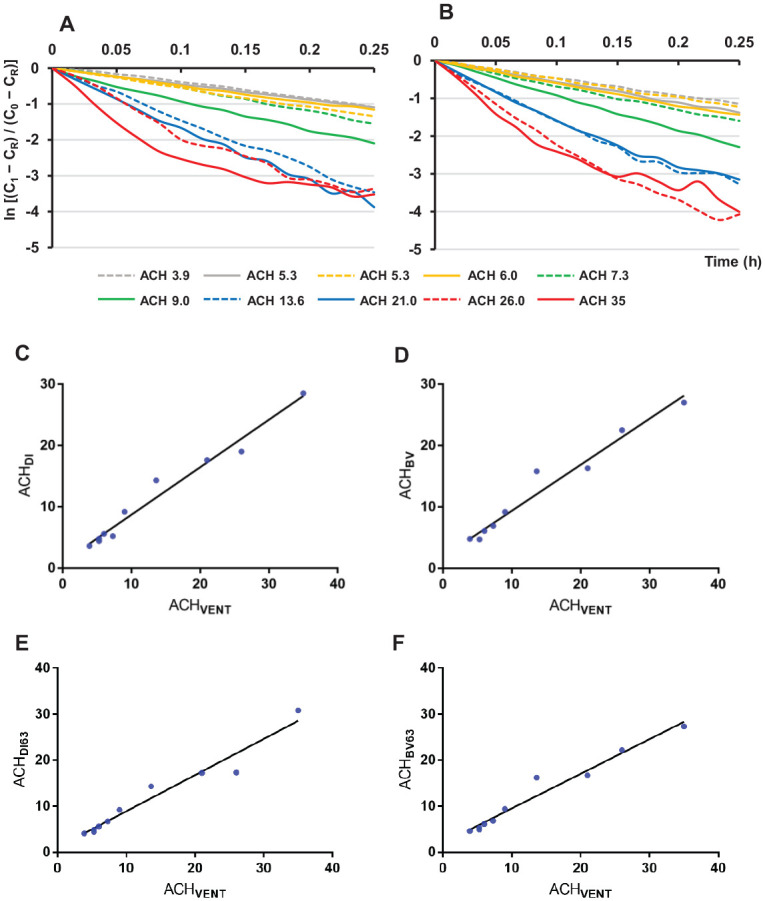
Correlations between ventilation rate and carbon dioxide (CO_2_) clearance in dental treatment rooms. (**A**) CO_2_ decay constants by dry ice and (**B**) CO_2_ decay constants by baking soda and vinegar. (**C**, **D**) Correlations between mechanical ventilation rates measure by airflow (ACH_VENT_) and ventilation rates measured by CO_2_ decay using dry ice (ACH_DI_) and baking soda and vinegar (ACH_BV_) in dental treatment rooms. Rooms with high mechanical ventilation rates showed a rapid decrease of CO_2_ concentrations over time (A, B). Both ACH_DI_ and ACH_BV_ are linearly correlated with ACH_VENT_ (C, D). (**E**, **F**) Correlations between ventilation rate by airflow (ACH_VENT_) and ventilation rates by time needed to reach 63% removal of excess CO_2_ generated using (E) dry ice (ACH_DI63_) and (F) baking soda and vinegar (ACH_BV63_).

Results of ventilation estimates by CO_2_ decay using dry ice or baking soda are shown in Table 2. CO_2_ decay curves demonstrate that CO_2_ levels decreased faster over time in rooms with high air change rate (Fig. 3A, B). ACH_DI_ values ranged from 3.6 to 28.5 (11.2 ± 8.4) and were highly correlated with the mechanical ventilation rate (*r* = 0.99, *P* < 0.0001) (Fig. 3C). ACH_DI_ was slightly lower than the mechanical ventilation rate (mean difference = 2.0, paired *t* = 2.32, *P* = 0.046). Similarly, ACH_BV_ ranged from 4.7 to 27.0 (11.8 ± 8.1) and also correlated highly with mechanical ventilation rate (*r* = 0.98, *P* < 0.0001) (Fig. 3D). There was no statistically significant difference between ACH_BV_ and the mechanical ventilation rate (mean difference = 1.4, paired *t* = 1.48, *P* = 0.174) or between ACH_BV_ and ACH_DI_ (mean difference = 0.59, paired *t* = 1.26, *P* = 0.239).

### Ventilation Rates by Time to 63% Removal of Excess CO_2_

Ventilation rates calculated by time needed to remove 63% of excess CO_2_ generated by dry ice or baking soda are presented in Table 2. ACH_DI63_ values ranged from 4.1 to 30.8 (11.5 ± 8.6) and were highly correlated with the mechanical ventilation rate (*r* = 0.98, *P* < 0.0001) (Fig. 3E). There was no statistically significant difference between ACH_DI63_ and mechanical ventilation rate (mean difference = 1.8, paired *t* = 1.93, *P* = 0.086).

Similarly, ACH_BV63_ ranged from 4.6 to 27.3 (11.9 ± 8.1) and correlated highly with the mechanical ventilation rate (*r* = 0.98, *P* < 0.0001) (Fig. 3F). There was no statistically significant difference between ACH_BV63_ and mechanical ventilation rate (mean difference = 1.3, paired *t* = 1.34, *P* = 0.213) or between ACH_BV63_ and ACH_DI63_ (mean difference = 0.50, paired *t* = 0.76, *P* = 0.467).

In Appendix Table 4, a Microsoft Excel template is provided that will allow dental care professionals to calculate the ventilation rate of their offices by inputting the values of peak CO_2_ level (C_S_), outdoor CO_2_ level (C_R_), and time (min) needed to reach 63% removal of excess CO_2_ generated by baking soda (Jimenez 2020).

## Discussion

Our findings affirmed that dental operatories with low ventilation rates and overcrowding facilitate CO_2_ accumulation. Ventilation can be measured by assessing natural or experimental buildup of CO_2_ levels in dental treatment rooms using a consumer-grade CO_2_ sensor. Ventilation rates in air change per hour could be accurately assessed by observing CO_2_ levels after a simple mixing of household baking soda and vinegar in dental settings. Time needed to remove 63% of excess CO_2_ generated by baking soda could be used to accurately calculate the ventilation rates with the help of a basic calculator.

Our findings show that CO_2_ level may consistently stay above 800 ppm in rooms with ventilation rates below 6 ACH, especially when 3 or more persons (including the patient who is not wearing a mask) are in the room during dental treatments. We observed that CO_2_ level stayed above 1,000 ppm and approached 1,600 ppm when 3 to 6 persons were in a room with 3.9 ACH in clinical teaching scenarios. High levels of CO_2_ indicate high concentrations of respiratory aerosols in the room. It is possible that these aerosols contain pathogens if the patient is not wearing a mask and is infected but asymptomatic or presymptomatic. Effective mitigation measures will be required in these rooms to improve air quality even without the ongoing infectious disease pandemics. Overcrowding should be avoided in rooms with poor ventilation. In dental operatories with ventilation rates higher than 10 ACH, the CO_2_ levels stayed consistently below 700 ppm in most cases with 3 persons in the room. Our data demonstrated a clear dependency of CO_2_ levels on number of persons in the room and the ventilation rate. CO_2_ level is a proxy for indoor air quality as it represents the fraction of rebreathed air, or the proportion of inhaled air that was exhaled by others in the same indoor environment. Although numerous epidemiological studies indicate CO_2_ begins to have negative health effects at 700 ppm and respiratory symptoms may occur when indoor CO_2_ is above 1,000 ppm (Azuma et al. 2018), our main concern is the concurrent accumulation of respiratory aerosols that may contain infectious disease pathogens. Numerous studies have shown that exhaled air from infected patients contains respiratory disease pathogens, including rhinovirus, influenza virus, and *Mycobacterium tuberculosis* (Fabian et al. 2009; Issarow et al. 2015; Lindsley et al. 2016; Yip et al. 2019). Patients with early stages of coronavirus disease 2019 (COVID-19) may release millions of severe acute respiratory syndrome coronavirus 2 (SARS-CoV-2) viral copies per hour in exhaled air (Qian et al. 2020). In a recent study that modeled factors associated with the spread of respiratory infectious disease in dental offices, CO_2_ levels were found to play the most important role in the risk of disease transmission. CO_2_ levels at 774 ppm were considered low risk, but those at or above 1,135 ppm may increase the risk of disease transmission in dental offices (Zemouri et al. 2020).

Ventilation rates varied greatly between rooms on the same air handling system due to variations in their sizes and locations and the addition of separate air exhaust fans in some rooms designated for nitrous oxide conscious sedation (Ren et al. 2021). Accurate measurements of ventilation rate in dental settings are important for risk assessment and for risk mitigation planning in an era of frequent infectious disease pandemics. Mechanical ventilation rate is usually assessed by quantifying the amount of outdoor air flowing into and out of an indoor space using highly sophisticated instruments operated by trained professionals (ASHRAE 2017). Technical barriers may have contributed to the scarcity of information regarding ventilation in dental settings. Besides direct airflow measurements, ventilation rate could be estimated using CO_2_ as a tracer gas. CO_2_ in an indoor space could be built up to a significantly higher level than in outdoor air, either through natural generation by the occupants or through experimental release of the gas (Cheng and Li 2014; Stuart et al. 2015; Batterman 2017). Analysis of steady-state CO_2_ levels or the rate of CO_2_ concentration decays, which are directly dependent on the outdoor airflow rate from the ventilation system, will allow an estimate of the ventilation rate of the indoor space. We found that modeling CO_2_ levels using equation (1) correlated reasonably well with mechanical ventilation but may either under- or overestimate the ventilation rate based on different assumptions of human CO_2_ generation rates. In comparison, CO_2_ concentration decay method relied on actual CO_2_ levels measured at the beginning and the end of a decay period and provided accurate assessments and better approximation to mechanical ventilation rates. CO_2_ concentrations in dental operatories could be built up to a level of about 1,500 to 2,500 ppm in 2 min using either dry ice or baking soda. CO_2_ decays could then be monitored using a CO_2_ sensor that logs data in 1-min intervals. Many affordable consumer-grade CO_2_ sensors are readily available and suitable for the purpose of observing CO_2_ level changes over a period of time. The CO_2_ sensor used in the present study was purchased online for $159 and appeared to be a reliable tool for measuring CO_2_ levels in dental settings.

We recommend that mitigation measures be taken for dental operatories that have ventilation rates below 15 ACH, which is required for procedure rooms in outpatient health care facilities by Centers for Disease Control and Prevention guidelines (Chinn et al. 2003). While in theory the most effective measure for air quality improvement in dental offices is to increase outdoor airflow rate through the ventilation system or through natural ventilation by opening doors and windows, such measures are severely limited by the weather or climate conditions. An effective alternative is to improve air filtration using upgraded filters in the ventilation system and portable air cleaners (PACs) equipped with high-efficiency particulate air (HEPA) filters (Ren et al. 2021).

In summary, this study showed that CO_2_ level in dental treatment rooms could be measured with a simple consumer-grade CO_2_ sensor and that ventilation rate could be determined by either natural or experimental buildup of CO_2_ levels in dental settings. Assessing CO_2_ levels will allow dental care professionals to conveniently and accurately calculate the ventilation rates in their offices and help them to devise an effective strategy for ventilation improvement.

## Author Contributions

Q. Huang, contributed to conception, design, data acquisition, and analysis, critically revised the manuscript; T. Marzouk, R. Cirligeanu, contributed to data acquisition, critically revised the manuscript; H. Malmstrom, E. Eliav, contributed to conception, design, and data interpretation, critically revised the manuscript; Y.-F. Ren, contributed to conception, design, data acquisition, analysis, or interpretation, drafted the manuscript. All authors gave final approval and agree to be accountable for all aspects of the work.

## Supplemental Material

sj-pdf-1-jdr-10.1177_00220345211014441 – Supplemental material for Ventilation Assessment by Carbon Dioxide Levels in Dental Treatment Rooms Click here for additional data file. Supplemental material, sj-pdf-1-jdr-10.1177_00220345211014441 for Ventilation Assessment by Carbon Dioxide Levels in Dental Treatment Rooms by Q. Huang, T. Marzouk, R. Cirligeanu, H. Malmstrom, E. Eliav and Y.-F. Ren in Journal of Dental Research
