# Supplementary material for: Ventilation Assessment by Carbon Dioxide Levels in Dental Treatment Rooms
Source: J Dent Res. 2021 May 11;100(8):810–6. doi: 10.1177/00220345211014441 (PMC8120146; doi:10.1177/00220345211014441)
Supplement: sj-pdf-1-jdr-10.1177_00220345211014441 – Supplemental material for Ventilation Assessment by Carbon Dioxide Levels in Dental Treatment Rooms [file sj-pdf-1-jdr-10.1177_00220345211014441.pdf]

Ventilation assessment by carbon dioxide levels in dental treatment rooms

Qirong Huang, Tamer Marzouk, Razvan Cirligeanu, Hans Malmstrom, Eli Eliav, Yan-Fang Ren
Eastman Institute for Oral Health, University of Rochester Medical Center, Rochester, New York

Supplemental materials:

Background: Risks of disease transmission in healthcare settings during infectious disease pandemics have consistently challenged dental care professionals in their efforts to maintain a safe environment for their staffs and patients. Dental care professionals have gained tremendous experiences in infection control from the ongoing HIV/AIDS pandemic by implementing universal or standard precautions against contact and droplet transmissions, but we are less confident in dealing with an infectious respiratory disease that may be transmitted through aerosol particles emitted by patients who have no overt symptoms. With mounting evidence that COVID-19 is transmissible through aerosols in an indoor environment (CDC 2020a; 2020b), additional preventive measures beyond the standard care using personal protective equipment (PPE) are essential to minimize risks and alleviate anxieties experienced by staff and patients due to uncertainties associated with a novel infectious disease pandemic.

Engineering controls through mechanical ventilation are important mechanisms to reduce the risks of airborne disease transmission in an indoor environment such as the dental offices. Though CDC recommends improving ventilation and air filtration in its guidance for dental settings during the COVID-19 pandemic (CDC 2020c), few information is available on how to assess the ventilation condition and what measures to take to achieve more effective engineering control of disease transmission in dental offices.

Using steady state CO_2 level during dental treatments to estimate ventilation rates: Ventilation rate estimates based on steady state CO_2 levels during two different treatment procedures at low (0.3 L/min) and high (0.46 L/min) levels of human CO_2 generation rates are presented in Supplemental Table 1 (Batterman 2017, Godwin et al 2003).

Appendix Table 1: Ventilation rate estimates based on steady state CO_2 levels

Rm #	Procedure	n	C_{SS}	C_R	ACH_{SS30}	ACH_{SS46}
**002**	Exam	4	1014	410	5.2	7.9
	Extraction	4	978	410	5.5	8.4
**003**	Extraction	4	960	410	5.9	9.0
	Extraction	4	923	410	6.3	9.7
**008**	Hygiene	2	673	435	4.4	6.7
	Hygiene	2	629	435	5.4	8.2
**012**	Hygiene	2	649	434	5.8	8.9
	Hygiene	2	632	434	6.3	9.7
**019**	Extraction	4	823	403	8.8	13.5
	Restorative	3	616	403	13.1	20.1
**021**	Implant	4	905	410	6.0	9.1
	Endo	4	926	410	5.7	8.8
**022**	Implant	6	1269	428	5.4	8.3
	Implant	5	1089	428	5.8	8.8
**031**	Hygiene	2	544	405	9.5	14.6
	Surgery	4	611	405	12.9	19.7
**032**	Surgery	3	584	404	15.8	24.2
	Exam	4	633	404	16.7	25.5
**033**	Exam	3	595	392	9.7	14.8
	Surgery	4	662	392	10.6	16.2
**Mean**	-	3.5	785.8	413.1	8.2	12.6
**SD**	-	1.1	206.8	14.0	3.8	5.8

Based on ventilation rate $ACH = 6 \times 10^4 n G_P / [V(C_{SS}-C_R)]$; n: number of persons in the room; V: volumetric size in m^3. C_{SS}: steady state CO_2 level in ppm; C_R: outdoor CO_2 level in ppm; ACH_{SS30}: ventilation estimate based on CO_2 generation rate G_P=0.3L/min per person; ACH_{SS30}: ventilation estimate based on CO_2 generation rate G_P=0.46L/min per person;

Appendix Table 2 could be used to roughly estimate the ventilation rate of the dental treatment rooms in 3 steps. First, measure the length and width of the room to get the area in square feet (ft^2). Second, determine the steady state CO_2 level during a dental treatment procedure that lasts more than 10 minutes as follows: with the dentist, dental assistant and the patient together in the room and without any person entering or leaving the room, read the CO_2 sensor readings 10 minutes into the procedure and record the next 5 readings at 1 min interval, add the 5 readings to get the sum and divide the sum by 5, the result is the steady state CO_2 level. Third, match the steady state CO_2 level to the closest number under the area column of your room size, the number in the ACH column in the same row is the ventilation rate estimate. For example, if the CO_2 level reaches a steady state level of about 1062 ppm during a dental treatment that lasted longer than 5 minutes with 3 persons in a room that is 110 ft^2 (10-ft W x11-ft L) in area, the ventilation rate is about 3 ACH. It will be about 6 ACH if the CO_2 level stays at about 761 ppm (Appendix Table 2).

Appendix Table 2: Steady state CO_2 levels and ventilation rate in air change per hour (ACH)*

ACH \ Area (ft^2)	100	110	120	130	140	150	160	170	180	190	200
**1**	2784	2567	2386	2234	2103	1989	1890	1802	1724	1655	1592
**2**	1592	1484	1393	1317	1251	1195	1145	1101	1062	1027	996
**3**	1195	1122	1062	1011	968	930	897	867	841	818	797
**4**	996	942	897	858	826	797	772	751	731	714	698
**5**	877	833	797	767	741	718	698	680	665	651	638
**6**	797	761	731	706	684	665	648	634	621	609	599
**7**	741	710	684	662	643	627	613	600	589	579	570
**8**	698	671	648	629	613	599	586	575	566	557	549
**9**	665	641	621	604	589	577	566	556	547	539	532
**10**	638	617	599	583	570	559	549	540	532	525	519
**11**	617	597	581	567	555	544	535	527	520	514	508
**12**	599	581	566	553	542	532	524	517	510	505	499
**13**	583	567	553	541	531	522	515	508	502	497	492
**14**	570	555	542	531	522	514	506	500	495	490	485
**15**	559	544	532	522	514	506	499	493	488	484	479

*Based on ventilation rate ACH = $6 \times 10^4 n G_P / [V(C_{SS}-C_R)]$, where number of persons in the room n=3, CO_2 generation rate per person G_P=0.3L/min, outdoor CO_2 level C_R=400 ppm, and V is the volumetric size of the room with ceiling height = 8-ft.

Protocol for assessing ventilation rates by CO_2 decays using dry ice: Outside air CO_2 level was first measured for 5 minutes near the air intake of the ventilation system outside the building before each experiment. To raise the peak CO_2 levels inside the dental treatment rooms to approximately 2000 ppm, 250g of dry ice was placed in a water bath and left in the room for two minutes. A small oscillating fan was used to keep the CO_2 well mixed in the room. CO_2 level was then measured at one-minute intervals using a consumer-grade CO_2 sensor (Aranet4, range 0-9999ppm, accuracy ±50ppm, SAF Tehnika, Riga, Latvia) for up two hours. The consumer-grade Aranet4 CO_2 sensor used in the current study was purchased at amazon.com in the US. It was recommended by the Federation of European Heating, Ventilation and Air Conditioning Associations (REHVA) for monitoring CO_2 levels in schools during the COVID-19 pandemic (REHVA 2020), and was found to be comparable to a research-grade LI-COR CO_2 sensor in accuracy and suitable for the time-response assessment in this study (Jimenez 2020).

Protocols for assessing ventilation rates by CO_2 decays using baking soda: We tested peak CO_2 values in the 10 treatment rooms after mixing baking soda with vinegar using a weight (g):volume (ml) ratio of 1:15 based on the molar masses of the reagents. We aimed at a peak level range of 1500 to 2000 ppm in rooms with various mechanical ventilation rates and with doors closed. We determined

that adding about 125g of baking soda (approximately 3/5 cup measure) to 1893 ml (a 64-oz bottle) of vinegar containing 5% acetic acid will elevate the CO_2 level in a typical dental treatment room (10 x 11 ft in area, 8 ft in ceiling height, or 880 ft^3 in volume) with a moderate ventilation rate (ACH_{VENT} = 4) to above 1500 ppm. For rooms that are significantly larger or having very high ventilation rates, one full cup measure (about 8 oz or 227g) of baking soda may be used with 3785 ml (a one-gallon jar) of vinegar containing 5% acetic acid.

Calculating ventilation rate using CO_2 level change after mixing baking soda and vinegar: Details of ventilation rates calculated by time needed to remove 63% of excess CO_2 generated by dry ice or baking soda are presented in Appendix Table 3.

Appendix Table 3: Ventilation rate estimates by time needed to remove 63% excess CO_2 released by dry ice or baking soda and vinegar

RM#	Method	C_R ppm	C_S ppm	C_E ppm	$C_{63\%E}$ ppm	t_2 min	$ACH_{T63\%}$ $60/t_2$
**002**	DI	403	3956	3553	1718	10.7	5.6
	BV	410	2800	2390	1294	9.9	6.1
**003**	DI	416	3552	3136	1576	12.3	4.9
	BV	412	1901	1489	963	12.3	4.9
**008**	DI	434	2459	2025	1183	9.0	6.7
	BV	399	2290	1891	1099	8.8	6.8
**012**	DI	427	3064	2637	1403	6.5	9.2
	BV	410	2340	1930	1124	6.4	9.4
**019**	DI	434	2901	2467	1347	2.0	30.8
	BV	399	1112	713	663	2.2	27.3
**021**	DI	434	4265	3831	1852	13.5	4.4
	BV	410	1782	1372	917	10.6	5.7
**022**	DI	427	4103	3676	1787	14.7	4.1
	BV	399	2474	2075	1167	13.0	4.6
**031**	DI	427	2066	1639	1033	3.5	17.2
	BV	416	2006	1590	1004	3.6	16.7
**032**	DI	427	2703	2276	1269	3.5	17.3
	BV	410	2139	1729	1049	2.7	22.2
**033**	DI	434	2530	2096	1210	4.2	14.3
	BV	432	1291	859	750	3.7	16.2

DI: dry ice. BV: baking soda and vinegar. C_R: outdoor CO_2 level. C_S: peak CO_2 level after CO_2 generation by dry ice or baking soda. C_E: excess CO_2 generated by dry ice or baking soda (C_S - C_R). $C_{63\%E}$: CO_2 level after 63% excess CO_2 is removed (C_S- 63%C_E). t_2: time (min) needed to reach $C_{63\%E}$. $ACH_{T63\%}$: ventilation rate in air change per air based on t_2

Appendix Table 4 is a dynamic template that will allow you to enter 3 values to get the ventilation rate in air change per hour (ACH) for your treatment rooms: 1, the peak CO_2 level (C_S), 2, the outdoor CO_2 level, and 3. Time needed to reach 63% removal of excess CO_2. (Fernstrom and Goldblatt 2013; Jimenez 2020; Nardell et al. 1991)

Appendix Table 4: Ventilation rate estimate using time needed to remove 63% excess CO_2 generated by baking soda and vinegar

CO_2 at peak	2800	ppm	B1. C_S = Peak CO_2 level C_S
CO_2 outdoors	400	ppm	B2. C_R = Outdoor CO_2 level C_R
Excess CO_2	2400	ppm	B3. C_E = Excess CO_2. C_E = C_S-C_R
After removal of 63% excess CO_2	1288	ppm	B4. $C_{63\%}$ = CO_2 level after removal of 63% C_E. $C_{63\%}$ = C_S - 63% C_E
Time needed to remove 63% excess CO	15.0	min	B5. Time to reach $C_{63\%}$ (Value in B4)
Ventilation rate	4.0	h^{-1}	B6. Air change per hour, or ACH
To calculate air change per hour, 3 values need to be entered into the above template:			
1. C_S: this is the CO_2 level when the measurement starts after CO_2 generation by mixing baking soda and vinegar for 2 minutes.			
2. Outdoor CO_2 level. You could use 400 ppm as an estimate for this value.			
3. Time needed to reach 63% removal of excess CO_2.			
You need to read the value in B5 and check the CO_2 sensor readings to get the time needed to reach 63% removal of excess CO_2.			

Application notes: It is important to point out that the setting for the current study is a postdoctoral dental training institution affiliated with an academic medical center, which may differ significantly in ventilation conditions from private dental practices that have a solo or a few dental practitioners. Ventilation conditions in different dental settings are largely unknown as the ventilation design of dental offices is not regulated as other outpatient healthcare facilities that are required to have 6 to 15 ACH by ASHRAE and CDC (Chinn and Sehulster 2003; Ninomura and Bartley 2001). Godwin and colleagues reported that ventilation rate was 1.12 ACH in dental operatories of a small dental clinic (2400 ft^2) (Godwin et al. 2003), which is significantly lower than the mean of 13 ACH in the present study but resembles more closely to the mean of 1.09 ACH in typical residential households in the US (Sherman and Matson 1997).

We consider that it is very important for every dental practitioner to be able to accurately assess the ventilation rate in their working environments. Epidemiological data showed that transmission of COVID-19 is almost exclusively an indoor phenomenon, with 99.97% of the transmissions occurring in an indoor environment (Qian et al. 2020). Airborne transmission through respiratory aerosols is increasingly recognized as a major driver for the COVID-19 pandemic (Morawska and Milton 2020; Noorimotlagh et al. 2020; Zhang et al. 2020). As essential healthcare providers, dental professionals work in the frontline during the pandemic and need to adopt measures to mitigate the risk of aerosol transmission in addition to droplet and contact precautions that have been the standard of infection control in dental offices (Harte 2010).

Our data showed that household baking soda ($NaHCO_3$) and vinegar (5% acetic acid) could be used to generate CO_2 in dental office to assess the ventilation rate by observing the CO_2 concentration decays using a CO_2 sensor and a basic calculator. This method will allow dental practitioners to reliably estimate the ventilation rate in their dental offices without expensive equipment and advanced technical skills. The test could be completed within 30 minutes in spaces with ventilation rate higher than 2 ACH but may take longer time if the ventilation is significantly below 1 ACH. We recommend to plan a two-hour observation time during off-hours with the building ventilation system operating in its normal setting.

Although ventilation rate in ACH could be calculated by fitting a linear regression line over time into the natural log scale of time-varying concentrations of CO_2 levels (equation #2), we found that a simplified method (equation #4) provided equally if not more accurate estimate of ventilation rate. As it is known that one complete air change replaces 63% of airborne contaminants with outdoor air (Fernstrom and Goldblatt 2013; Jimenez 2020; Nardell et al. 1991), ventilation rate could be easily calculated using the time needed to remove 63% of excess CO_2. For example, assuming outdoor CO_2 level is 400ppm, and peak CO_2 level is 1500ppm after mixing baking soda with vinegar inside the dental office for 2 minutes, excess CO_2 inside the room will be 1500–400=1100ppm at peak. The CO_2 level that represents 63% removal of excess CO_2 is therefore 1500–63% x1100=807ppm. If it takes 15min for CO_2 level to reach 807ppm from the peak of 1500ppm, ventilation rate will be 60/15=4 ACH; and if it takes 2 hours for the CO_2 level to reach 807ppm, it will be 60/120 = 0.5 ACH. This method will allow dental care professionals to accurately estimate ventilation rate using a simple calculator.

Abbreviations:

ACH	air change per hour
ACH_{VENT}	air change per hour measured by an air velocity sensor integrated in an airflow balancing hood at air supply vent or air exhaust vent
ACH_{SS30}	air change per hour calculated from steady state CO_2 levels at a CO_2 generation rate of 0.30L/min

ACH_{SS46}	air change per hour calculated from steady state CO_2 levels at a CO_2 generation rate of 0.46L/min
ACH_{DI}	air change per hour calculated by CO_2 clearance using the dry ice method
ACH_{BV}	air change per hour calculated by CO_2 clearance using the baking soda and vinegar method
ACH_{DI63}	air change per hour calculated from time needed to remove 63% excess CO_2 generated by dry ice
ACH_{BV63}	air change per hour calculated from time needed to remove 63% excess CO_2 generated by baking soda and vinegar

References:

C.D.C. How covid-19 spreads. 2020a. [accessed 2021 Feb. 4]. https://www.cdc.gov/coronavirus/2019-ncov/prevent-getting-sick/how-covid-spreads.html.

C.D.C. Scientific brief: Sars-cov-2 and potential airborne transmission. 2020b. [accessed 2021 Feb. 4]. https://www.cdc.gov/coronavirus/2019-ncov/more/scientific-brief-sars-cov-2.html.

C.D.C. Uidance for dental settings - interim infection prevention and control guidance for dental settings during the covid-19 pandemic. 2020c. [accessed 2021 Feb. 4]. https://www.cdc.gov/coronavirus/2019-ncov/hcp/dental-settings.html.

Chinn RYW, Sehulster L. 2003. Guidelines for environmental infection control in health-care facilities; recommendations of cdc and healthcare infection control practices advisory committee (hicpac).

Fernstrom A, Goldblatt M. 2013. Aerobiology and its role in the transmission of infectious diseases. J Pathog. 2013:493960-493960.

Godwin CC, Batterman SA, Sahni SP, Peng CY. 2003. Indoor environment quality in dental clinics: Potential concerns from particulate matter. Am J Dent. 16(4):260-266.

Harte JA. 2010. Standard and transmission-based precautions: An update for dentistry. J Am Dent Assoc. 141(5):572-581.

Jimenez, j-l. How to quantify the ventilation rate of an indoor space using an affordable co2 monitor. 2020. [accessed 2021 Feb. 4]. https://medium.com/@jjose_19945/how-to-quantify-the-ventilation-rate-of-an-indoor-space-using-a-cheap-co2-monitor-4d8b6d4dab44.

Morawska L, Milton DK. 2020. It is time to address airborne transmission of coronavirus disease 2019 (covid-19). Clin Infect Dis. 71(9):2311-2313.

Nardell EA, Keegan J, Cheney SA, Etkind SC. 1991. Airborne infection. Theoretical limits of protection achievable by building ventilation. Am Rev Respir Dis. 144(2):302-306.

Ninomura P, Bartley J. 2001. New ventilation guidelines for health-care facilities. ASHRAE Journal,. 43(6):29-32.

Noorimotlagh Z, Jaafarzadeh N, Martínez SS, Mirzaee SA. 2020. A systematic review of possible airborne transmission of the covid-19 virus (sars-cov-2) in the indoor air environment. Environ Res. 193:110612.

Qian H, Miao T, Liu L, Zheng X, Luo D, Li Y. 2020. Indoor transmission of sars-cov-2. Indoor Air.

Sherman MH, Matson N. 1997. Residential ventilation and energy characteristics. ASHRAE Trans. 103:717-730.

Zhang R, Li Y, Zhang AL, Wang Y, Molina MJ. 2020. Identifying airborne transmission as the dominant route for the spread of covid-19. Proc Natl Acad Sci U S A. 117(26):14857-14863.

Correspondence to:

Yanfang Ren, DDS, PhD, MPH
Professor
Eastman Institute for Oral Health
University of Rochester Medical Center
625 Elmwood Ave, Rochester, New York 14620, USA
Yanfang_ren@urmc.rochester.edu
Phone: +1 585 273 5588
